# High DNA Methyltransferase *DNMT3B* Levels: A Poor Prognostic Marker in Acute Myeloid Leukemia

**DOI:** 10.1371/journal.pone.0051527

**Published:** 2012-12-10

**Authors:** Sandrine Hayette, Xavier Thomas, Laurent Jallades, Kaddour Chabane, Carole Charlot, Isabelle Tigaud, Sophie Gazzo, Stéphane Morisset, Pascale Cornillet-Lefebvre, Adriana Plesa, Sarah Huet, Aline Renneville, Gilles Salles, Franck Emmanuel Nicolini, Jean-Pierre Magaud, Mauricette Michallet

**Affiliations:** 1 Service d’Hématologie Biologique, Centre Hospitalier Lyon-Sud, Pierre-Bénite France, Hospices Civils de Lyon, Lyon, France; 2 UMR5239 Pathologies des cellules lymphoïdes, Université Claude Bernard, Lyon, France; 3 Service d’Hématologie Clinique Centre Hospitalier Lyon-Sud, Pierre-Bénite France, Hospices Civils de Lyon, Lyon, France; 4 Laboratoire d'Hématologie, CHU, Reims, France; 5 Centre de Biologie-Pathologie Laboratoire d'Hématologie, CHRU de Lille, Lille, France; RWTH Aachen University Medical School, Germany

## Abstract

It has been recently shown that DNA methyl transferase overexpression is correlated with unfavourable prognosis in human malignancies while methylation deregulation remains a hallmark that defines acute myeloid leukemia (AML). The oncogenic transcription factor *EVI1* is involved in methylation deregulation and its overexpression plays a major role for predicting an adverse outcome. Moreover, the identification of DNMT3A mutations in AML patients has recently been described as a poor prognostic indicator. In order to clarify relationship between these key actors in methylation mechanisms and their potential impact on patient outcomes, we analysed 195 *de novo* AML patients for the expression of *DNMT3A*, *3B* (and its non-catalytic variant *3B_NC_*) and their correlations with the outcome and the expression of other common prognostic genetic biomarkers (*EVI1, NPM1, FLT3ITD/TKD* and *MLL*) in adult AML. The overexpression of *DNMT3B/3B_NC_* is (i) significantly correlated with a shorter overall survival, and (ii) inversely significantly correlated with event-free survival and *DNMT3A* expression level. Moreover, multivariate analysis showed that a high expression level of *DNMT3B/3B_NC_* is statistically a significant independent poor prognostic indicator. This study represents the first report showing that the overexpression of *DNMT3B/3B_NC_* is an independent predictor of poor survival in AML. Its quantification should be implemented to the genetic profile used to stratify patients for therapeutical strategies and should be useful to identify patients who may benefit from therapy based on demethylating agents.

## Introduction

Methylation-specific gene alteration is the major mechanism involved in inappropriate gene activation or silencing in leukemic cells and has been shown to be a universal feature occurring in all acute myeloid leukemia (AML) patients [1], [2]. DNA methyltransferases (DNMTs) are the main key effectors of DNA methylation by catalysing the transfer of a methyl group from the ubiquitous methyl donor S-adenosyl methionine to the 5′-position of cytosine residing in the dinucleotide sequence cytosine–guanine [3], [4]. At least three prototype-related structure DNMTs are known. DNA methyltransferase 3 (DNMT3A, DNMT3B) are thought to act as *de novo* DNMTs mostly implicated in somatic alterations [5]. DNMT3A is particularly required for the methylation of imprinted and single copy genes while DNMT3B is specialized in the methylation of pericentric satellite repeats [6], [7], [8]. Isoforms of DNMT3B can be divided into those who do not alter catalytic activity of DNMT (3B1, 3B2, 3B6) and others (3B3, 3B7, 3B8) may be inactive in catalysis [9] but could act as a rheostat in modulating DNMT3B and/or 3A [10], [11], [12]. The mechanism(s) by which cancer cells acquire alteration in DNA methylation is unknown but aberrant transcription of the *DNMT3B* gene is frequent [13]. DNMT3B7, a truncated non catalytic DNMT3B isoform particularly expressed in human tumors, has been shown to accelerate lymphomagenesis, increase chromosomal rearrangements and shows more locus specific perturbations in DNA methylation patterns in mice when *Dnmt3b7* transgenic mice are bred with *Eµ-Myc* transgenic mice [14]. Transcriptional activation of the human *EVI1* (*Ecotropic Virus Integration site 1*) gene located on 3q26.2, has been reported in up to 10% of AML patients and is an independent indicator of adverse prognosis [15], [16], [17]. Although most patients with 3q26 rearrangements (inv(3)(q21q26.2)/t(3,3)(q21;q26.2) overexpress *EVI1 (EVI1+)*, its level, through unknown mechanisms, is also elevated in about 10% of AML patients with no 3q aberrations [18]. EVI1 functions as a transcription repressor complex recruiting diverse proteins involved in chromatin remodelling.

High levels of EVI1 are associated with aberrant epigenetic signatures containing differentially hypermethylated genes, with an overrepresentation of EVI1 binding sites in their promoters [19]. DNMT3A mutations that abrogate its enzymatic activity are relatively common in *de novo* AML (22%) and in myelodysplasic syndromes (8%) [20], [21]. Their identification in AML patients has been recently described as a poor prognostic marker associated with disease progression and poor survival [22], [23], [24], [25]. Moreover, DNMT3A was found to be highly expressed in primary *EVI1+* AMLs as compared to other AML and a direct recruitment of DNMT3A and 3B by EVI1 has recently been demonstrated [1], [19]. The deregulation of DNMT3B expression clearly contributes to tumorogenesis and tumor suppressor gene hypermethylation [26]. In addition, a high expression level of DNMT*s* and their variants has been reported and described as a poor prognostic marker in various malignancies [27], [28].

Overall, these data provide a rationale that has prompted us to analyse a series of 195 *de novo* AML patients in order to establish at first time, the relationships between these strong mediators of gene expression (*DNMT3A/B* and *EVI1*) and other well-known biomarkers such as Nucleophosmin (*NPM1*), fms-like tyrosine kinase-3 (*FLT3*) mutations, partial tandem duplications of mixed-lineage leukemia gene (*MLL/PTD*) and *HOXA9* expression level. We studied in a second time their effect on survival outcomes.

## Materials and Methods

### Patients

#### Ethic statement

Written informed consent was obtained from all patients and the procedures followed were in accordance to the Helsinki declaration as revised in 2008. Samples were stored in the Biological Ressource Center Bank according to “the Comité de Protection des Personnes”. The review board protocol of the Hospices Civils de Lyon approved this study.

**Figure 1:**
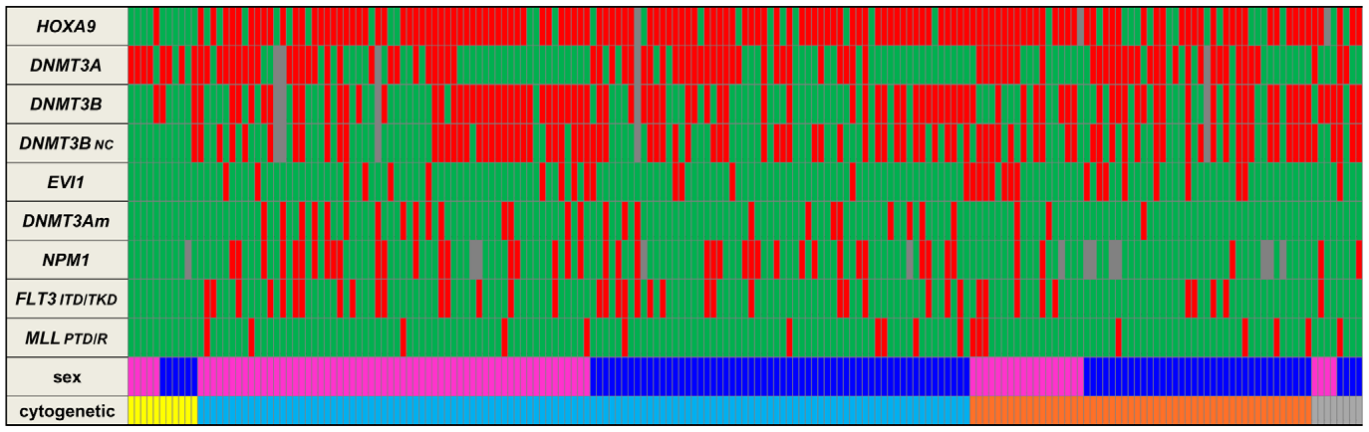
Genetic profile of each patient according to cytogenetic subgroups. Horizontal lines represent molecular markers, (red: mutated or overexpressed; green: wild-type or normal expression level; grey: not available data). One individual patient (195) in each column according to their cytogenetic subgroup (yellow: favourable karyotype; blue: intermediate karyotype; orange: unfavourable karyotype; grey: failed karyotype) and their gender (pink: female; dark blue: male).

Samples from 195 consecutive newly diagnosed AML patients (excluding acute promyelocytic leukemia) admitted at the Hematology department of Lyon University Hospital and treated onto clinical trials were referred to our laboratory between September 2002 and April 2011 (Table 1). AML was diagnosed according to the French-American-British (FAB) and World Health Organization classification of tumours criteria [29], [30]. There were 88 females and 107 males. Median age was 53 (range: 18–73 years). The main patient characteristics at diagnosis of AML are shown in Table 1 and in Figure 1. Induction consisted in one cytarabine- and anthracycline-based chemotherapy course (±salvage therapy). Patients achieving complete remission (CR) received then consolidation courses according to the trial in which they were included. Fifty-one patients with an HLA-compatible donor received allogeneic hematopoietic stem cell transplantation in first remission.

**Table 1:** Characteristics of the patients.

Variable	whole serie		*EVI1+*			*HOXA9+*			*DNMT3Am*			*DNMT3A+*		*DNMT3B+*			*DNMT3B_NC_+*			
	N	%		N	%		N	%		N	%	N	%		N	%		N	%	
(pos/neg/all)	195			32/163/195	16		100/93/193	25		29/166	15	93/99/192	49		101/90/191	53		97/94/191	51	
Age (years) Median	53 (18–73)	/		55 (18–70)	/		51 (20–73)	/		49 (28–67)	/	50 (18–73)	/		51 (18–73)	/		51 (18–71)	/	
Sex M/F	107/88	55		15/17	47		51/49	51		12/17	41	55/38	58		54/47	59		54/43	56	
Age <60 years	141	72		19	59		76	76		24	83	67	71		73	72		70	72	
Age ≥60 years	54	28		13	41		24	24		5	17	26	29		28	28		27	28	
M0/M1/M2	77	39		11	34		34	34		9	31	47	50		33	32		36	37	
M4/M5	74	38		11	34		48	48		14	48	28	30		40	40		34	35	
M6/M7	10	5		7	22		2	2		1	3	7	7		5	1		8	8	
8UC	34	18		3	9		16	16		5	17	11	12		23	23		19	20	
*NPM1*(pos/neg/all)	44/139/183	24		1/30/31	3		54/39/93	54		18/10/28	64	18/71/89	19		25/71/96	26		23/70/93	25	
*FLT3ITD/TKD*(pos/neg/all)	40/154/194	21		4/28/32	13		32/68/100	32		11/18/29	38	23/70/93	24		24/77/101	23		23/74/97	24	
*MLL PTD/R*(pos/neg/all)	19/175/195	10		6/26/32	19		16/84/100	16		3/26	10	9/84/93	10		14/87/101	14		12/85/97	4	
(1) K. Favourable	11	6		0	0		1	1		0	0	8	9		2	2		1	1	
(2) K. intermediate	121(93NC-AML)	61		16	50		67	67		26	90	53	57		64	63		60	62	
(3) K. Unfavourable	54	28		15	47		27	27		3	10	29	31		28	28		30	31	
(4) K. Failure	9	5		1	3		5	5		0	0	3	3		7	7		6	6	

F: female, M: male, pos: positive cases, neg: negative cases; M0 to M7: according to the French-American-British (FAB) diagnosis; 8UC (unclassified AML); nucleophosmin mutations (*NPM1*), fms-like tyrosine kinase-3 internal tandem duplications and tyrosine kinase domain mutations (*FLT3ITD/TKD*), mixed-lineage leukemia gene partial tandem duplications and rearrangements (*MLL PTD/R*); K: karyotype; CK complex karyotype (more than 3 abnormalities), NC-AML: Normal Cytogenetic Karyotype acute myeloid leukemia. To establish normal cytogenetic at least 20 metaphase cells from diagnostic bone marrow had to be evaluated and the karyotype had to be found normal in each mitosis. N: number of cases; +: positive cases; *DNMT3Am*: stands for mutated *DNMT3A; DNMT3B_NC_*: stands for non-catalytic *DNMT3B.*

### Cytogenetic Analyses

Cytogenetic R and G-banding analyses were performed according to standard methods. The definition of a cytogenetic clone and descriptions of karyotypes followed the International System for Human Cytogenetic Nomenclature. To be considered cytogenetically normal, at least 20 metaphases from bone marrow sample at diagnosis had to be evaluated. Abnormalities were categorized and classified into 3 categories (favourable, intermediate, and unfavourable) according to the British Medical Research Council’s classification [31]. In the overall cohort of 195 patients, 9 cytogenetic analyses failed and 93 normal cytogenetic (NC) karyotypes were identified in at least 20 metaphase cells evaluated from diagnostic bone marrow of each patient (Table 1).

### Molecular Assessments

#### Mutation and quantification of classical biomarkers

The fms-like tyrosine kinase-3 internal tandem duplications (*FLT3-ITD*), tyrosine kinase domain mutations (*FLT3-TKD*), nucleophosmin mutations (*NPM1*), mixed-lineage leukemia gene partial tandem duplications (*MLL/PTD*) and *Ecotropic Viral Integration Site 1* gene (*EVI1/1D*) expression were detected as previously described [32], [33], [34], [35].

#### RT/RQ-PCR of DNMT (3A/3B and 3BNC) and HOXA9 mRNA

Reverse transcription (RT) was performed as previously described [36] for 195 AML patients and 11 normal bone marrow donors. The targeted sequence for DNA corresponded to the mRNA of the two *DNMT* genes *DNMT3A*: ENST00000321117; *DNMT3B*: ENST00000328111 and *3B_NC_*: ENST00000456297. For ease and because all catalytic *(3B1, 3B2, 3B6)* and non-catalytic *(NC: 3B3, 3B7, 3B8)* DNMT3B spliceoforms showed respectively the same 3′ end nucleotide sequences, we used the term *3B* and *3B_NC_* for each subgroup. *DNMT (3A, 3B and 3B_NC_)* and *HOXA9* transcripts were amplified on the same cDNA using primers depicted in Table 2 and using Universal ProbeLibrary (UPL#3, FAM-MGB probe) according to the manufacturer’s instructions (Roche Diagnostics, Mannheim, Germany). Analysis were performed by comparative Ct method of relative quantification giving the amount of target normalized to an endogenous reference for RNA quality (namely, the *ABL* gene) as it was previously shown to be the most adequate for quantitative analysis in AML [37].

**Table 2:** Sequences of the different primers and probes.

GENE	Forward 5′-3′	Reverse 5′-3′	Probe 5′-3′
***HOXA9*** **RQ-PCR**	AAA ACA ATG CTG AGA ATG AGA GC	TAT AGG GGC ACC GCT TTT T	UPL: #3
***DNMT3B*** ** RQ-PCR**	GGT-GCA-CTG-AGC-TCG-AAA-G	AAG-AGG-TGT-CGG-ATG-ACA-GG	UPL: #3
***DNMT3B_NC_*** ** RQ-PCR**	TAC-CCG-GGA-TGA-ACA-GGA-T	AAG-AGG-TGT-CGG-ATG-ACA-GG	UPL: #3
***DNMT3A:*** **HRM/RQ PCR**	TGG-TGC-ACT-GAA-ATG-GAA-AG	ACT-GGC-ACG-CTC-CAT-GAC	UPL: #3
***DNMT3A*** ** HRM**	TGG-TGC-ACT-GAA-ATG-GAA-AG	GTT-TGC-CCC-CAT-GTC-CCT-TA	
***DNMT3B*** ** HRM**	GGT-GCA-CTG-AGC-TCG-AAA-G	GGC-TTG-GGG-CCT-GGC-TGG-AA	
DNMT3F^7–8^	GAG-TAC-GAG-GAC-GGC-CGG-GGC		
DNMT3F^8^	GCA-TTG-GGG-AGC-TGG-TGT-GG		
DNMT3AF^11–12^	GCC-GGA-ACA-TTG-AGG-ACA TC		
DNMT3AR^14–15^	GCA-AAA-GCA-CCT-GCA-GCA-GT		
DNMT3AR^16–17^	AGC-ACC-AGG-AGC-CCT-GTA-GCG		
DNMT3AF^16–17^	GCT-ACA-GGG-CTC-CTG-GTG-CT		
DNMT3AR^23INT^		GTT-TGC-CCC-CAT-GTC-CCT-TA	
DNMT3AR^23^		GCT-GAT-ACT-TCT-CTC-CAT-CCT	

UPL: #3; FAM - MGB probes from the Universal ProbeLibrary N° 04685008001 (ROCHE).

#### Mutational status of DNMT3A exon 23 in 195 AML patients

**Figure 2:**
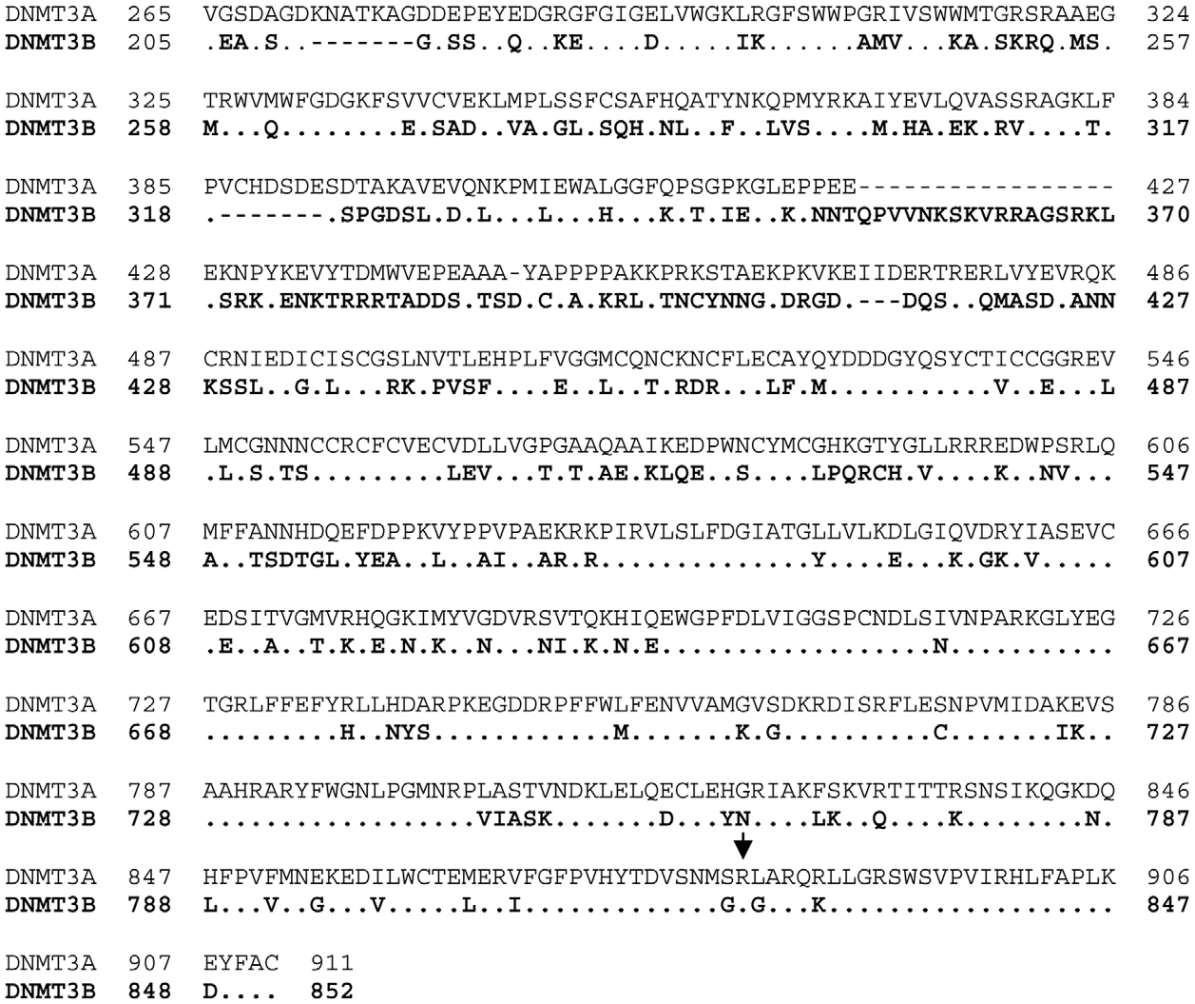
Alignment of DNMT3A with DNMT3B (in bold). The mutation site R882 of DNMT3A (corresponds to R823 in DNMT3B, which has been found mutated in ICF syndrome) is indicated by an arrow. Pairwise with dots stands for amino acid identities and dashes for gaps. Identities = 399/665 (60%), Positives = 489/665 (74%), Gaps = 35/665 (5%).

The screening of *DNMT3A* mutations (focused on exon 23 previously recognized as mostly mutated in AML) was performed by polymerase chain reaction (PCR) and High Resolution Melt analysis (HRM). PCR reactions were performed in a 20 µl final volume containing 5 µl cDNA and 0.3 µM *DNMT3A* primers (Table 2) with LC480 HRM master mix (Roche), containing 3.2 mM MgCl2 and Resolight© as nucleotide binding dye. Amplification was performed by 45 cycles of 95°C for 10 sec, 60°C for 15 sec and 72°C for 15 sec followed by a melt according to manufacturer instructions using a LightCycler 480 instrument (Roche Applied Sciences). HRM analysis is a suitable method in routine laboratories for the detection of the DNMT3A mutations located between amino acids V867 and R891 and especially for the most common mutation R882H/C with a 2.5%-sensitivity (Figure S1). All mutations were confirmed on another RT-PCR product and by direct sequencing. HRM analysis of *DNMT3B* catalytic region, highly similar to DNMT3A (Figure 2), were performed as described for the *DNMT3A* except for the use of primers from *DNMT3B* (Table 2).

#### Mutational status of DNMT3A (exon 8 to exon 23) in EVI1+ patients

In order to verify total absence of DNMT3A mutation in functional domain between exon 8 and exon 23 from 32 AML patients showing overexpression of *EVI1* (Table 1), a first round of PCR was performed on cDNA (5 µl), using primers DNMT3AF^7–8^ and DNMT3AR^23^ (Table 2). Thermocycling conditions used were 3 minutes at 94°C followed by 35 cycles of denaturation at 94°C for 1 minute, annealing at 60°C for 1 minute, extension at 68°C for 4 minutes, and a final extension step at 68°C for 10 minutes. Two nested PCR were performed using one fiftieth of the first PCR product described above, with the same thermocycling conditions and with forward (DNMT3AF^8^ and DNMT3AF^11–12^) and reverse (DNMT3AR^16–17^ and DNMT3AR^23INT^) primers, respectively (Table 2). Amplicons (1078 and 1297 bp) were directly sequenced in both strands using PCR primers and DNMT3AR^14–15^ and DNMT3AF^16–17^ depicted in Table 2. All mutations were confirmed on another RT-PCR product. Moreover, absence of mutations (identified at diagnosis) was verified on complete remission samples whenever available.

### Statistical Analyses

Complete remission (CR) was defined according to Cheson’s criteria [38]. Overall survival (OS) was calculated from the date of diagnosis until the date of death. Event-free survival (EFS) was measured from the date of diagnosis until the date of the first event (morphological relapse or death). Comparisons of patients' characteristics (covariates) were performed using the Fisher’s exact test for categorical variables, the Mann-Whitney U-test for continuous variables and by Spearman's rank correlation for quantitative variables. Quantitative variables as expression levels of *DNMT3A, 3B, 3B_NC_*, *HOXA9* and *EVI1* were also analysed as binary variables using median expression levels (*DNMTs* and *HOXA9*) or delta CT method (*DCTEVI1-ABL*<7) as *cut off* point in AML patients. OS and EFS rates were estimated by the Kaplan-Meier method and compared using the log-rank test. Covariates tested in multivariate Cox models were sex, age, *DNMTs, HOXA9, EVI1* expressions, cytogenetic risk group defined as previously described [39]. A p value<.05 was considered as statistically significant. In all univariate and multivariate analyses provided, the patients were censored at the time of transplantation but the same analyses were performed in the overall population without censoring allografted patients. All statistical analyses were performed in the normal karyotype subgroup (93 patients). Clinical data for first remission, overall survival and event-free survival analyses were available for all patients.

## Results

### Expression Analysis of *DNMT3A, 3B, 3B_NC_* and Correlation with other Biomarkers

**Figure 3:**
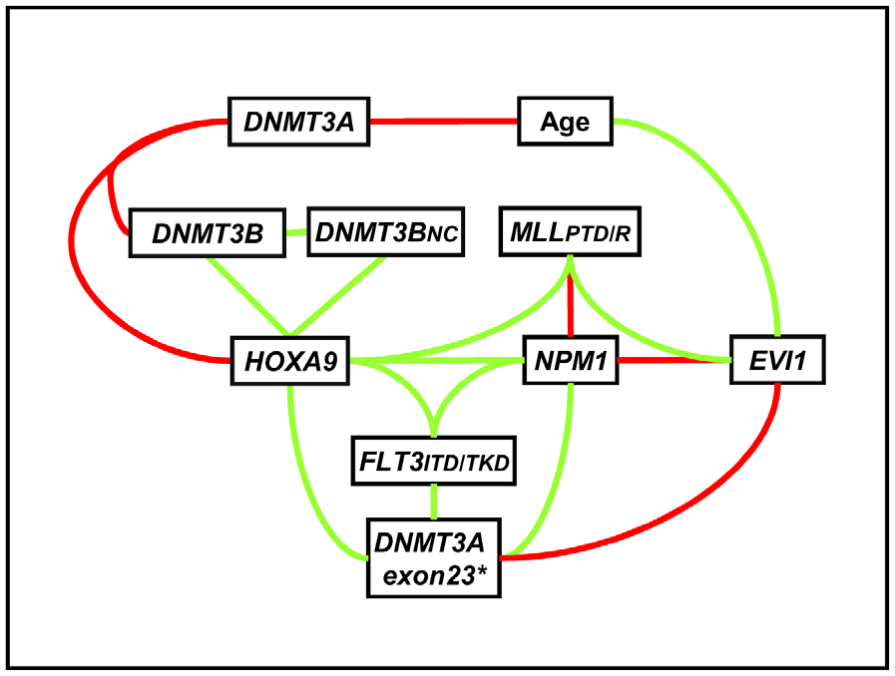
Statistically significant relationships between biomarkers. Graphical representation of significant Spearman's rank correlation Mann-Whitney U-test and/or Fisher’s exact test between two quantitative, quantitative/binary and binary variables;. Green lines significant positive relationships between the two variables tested; red lines: inversely related. Significant p values (<0.05). DNMT3A exon 23*: mutations in exon 23 of DNMT3A.

Among the whole population, Spearman's rank order correlation showed that *DNMT3A* expression was higher in young patients (p = 0.019; for<60 years) and in patients with low levels of *DNMT3B* (p = 0.002) and *HOXA9* (p = 0.005) whereas *DNMT3B* was highly related to *3B_NC_* (p<0.0000) and *HOXA9* expression (p = 0.0003). These results were confirmed using Mann-Whitney U-test and Fisher’s exact test (two tailed) when categorical variables were used for statistical analysis (Figure 3). *EVI1* overexpression was significantly related to AML patients with *NPM1* wild type status (p = 0.001) and to *MLL* abnormalities duplications (p = 0.039) or rearrangements (p = 0.001). Conversely to *EVI1*, *HOXA9* overexpression was more frequently found in *NPM1* (p<0.0000) and *FLT3-ITD/TKD* mutated patients (p = 0.0004; Figure 3). It should be noted that no correlation was observed at the transcriptional level between *EVI1* and *(i) DNMT3A* (p = 0.38), (ii) *DNMT3B* (p = 0.5) or (iii) *DNMT3B_NC_* (p = 0.27).

### *DNMT3* Mutation Analysis

Twenty-six mutated patients (13%) were identified in DNMT3A exon 23 and were confirmed by direct sequencing. Furthermore, HRM method allowed the identification of two new mutations onto amino acids R882 (R882S) and Q886 (Q886E). All new mutations have been verified on another RT-PCR product. Moreover, the Q886E mutation was not found in an available remission sample before transplantation which can invalidate the hypothesis of a polymorphism (data not shown). Morphologic data are summarized in Table 1. A slight association was found with monocytic differentiation (FAB M5 p = 0.04) but not confirmed towards myelomonocytic differentiation (FAB M4/M5; p = 0.27). As previously described [40] *DNMT3A* exon 23 mutations were mostly distributed in the normal karyotype category (p = 0.01) and exclusively in the intermediate cytogenetic risk group, with a strong association with *NPM1* mutations (p<0.000), *HOXA9* expression (p = 0.001) and *FLT3* mutations (p = 0.049) and inversely related to *EVI1* expression (p = 0.008). No *DNMT3A* exon 23 mutations were detected in high risk patients, particularly in *EVI1+* AML patients. Entire exon 8 to exon 23 sequencing of *DNMT3A* detected 3 further mutations in DNMT3A catalytic domain (V636E; D702G; R736C).

In order to screen this domain in DNMT3B, HRM analysis was performed from 137 patients’ samples. No change was observed in the catalytic active variant of DNMT3B (amino acids 808–836) and especially at amino acid R823 of DNMT3B, one of the most common mutated amino acids involved in hereditary syndrome characterized by ICF and which corresponds to the amino acid R882 of DNMT3A (Figure 2).

### Impact of HOXA9, EVI1,and DNMTs Expression Levels on Overall Survival (OS) and Event-free Survival (EFS)

**Figure 4:**
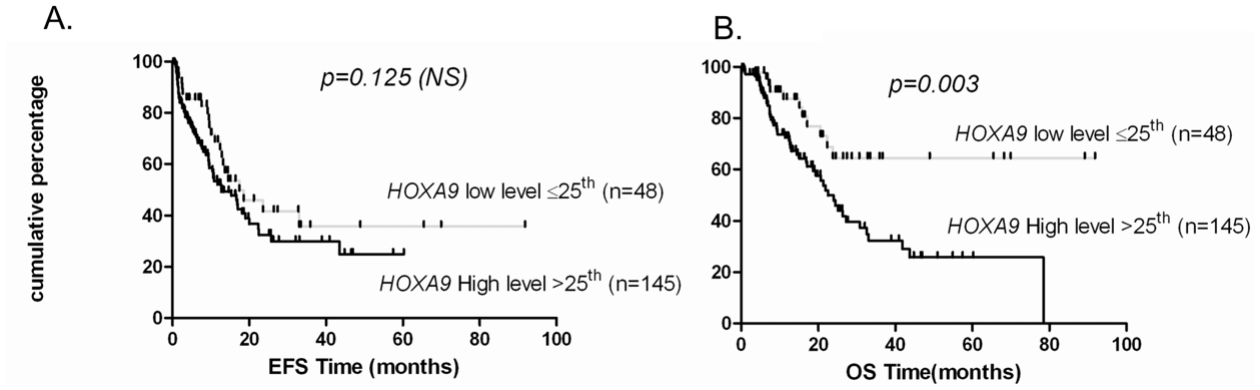
Low HOXA9 expression associates with better survival outcome. Kaplan Meier analysis of two groups with expression levels above or below the cut-off at the first quartile for HOXA9 expression. (A) Event free survival (EFS) and (B) Overall survival (OS) were assessed in 193 patients.

*HOXA9* overexpression had no prognostic impact whereas patients with low *HOXA9* expression level had a better OS than patients with higher *HOXA9* expression level (p = 0.003; OS median not reached Vs 23 months) (Figure 4). *HOXA9* expression levels had no prognostic impact on EFS.

**Figure 5:**
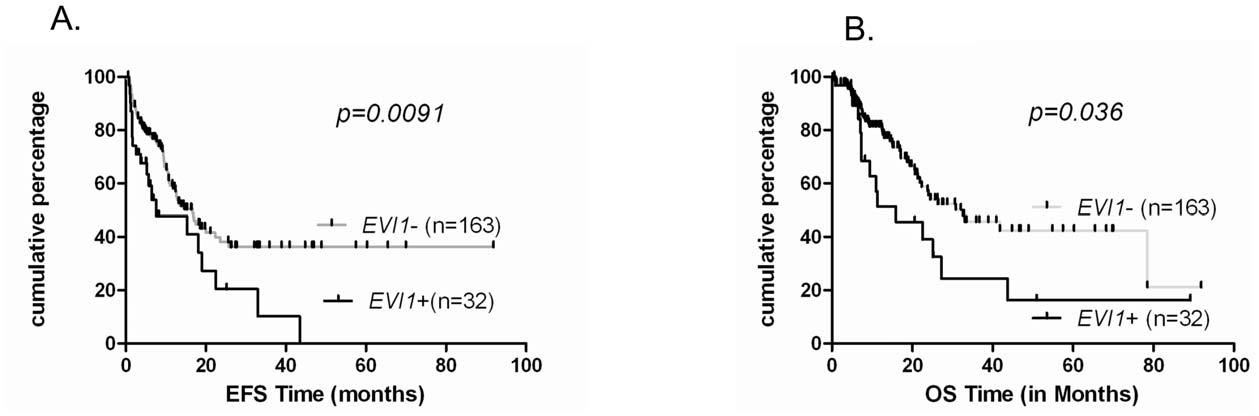
High *EVI1* expression associates with poor survival outcome. Kaplan Meier analysis of two groups with expression levels above or below the cut-off as described in method section for *EVI1* (delta CT<7). EFS (A) and OS (B) were assessed in 195 patients.

Patients who showed an overexpression of *EVI1* had significant lower EFS and OS (Figure 5) and when we stratified on age, this observation was more important in young patients (EFS: p = 0.002 and OS: p = 0.037). It has been recently described that patients who showed a total lack of *EVI1* expression might have a good prognosis [41], In our series, patients with absolutely no *EVI1* expression did not show better survival than those with basal *EVI1* expression, even in patients below 60 years (data not shown).

**Figure 6:**
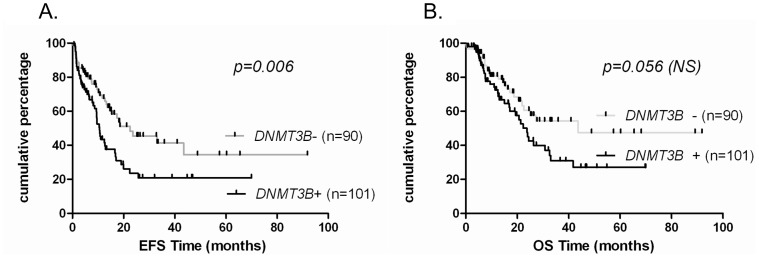
High *DNMT3B* expression associates with poor event free survival. Kaplan-Meier analysis of two groups with expression levels above or below the cut-off as described in method section for *DNMT3B* EFS (A) and OS (B) were assessed in 191 patients.

**Figure 7:**
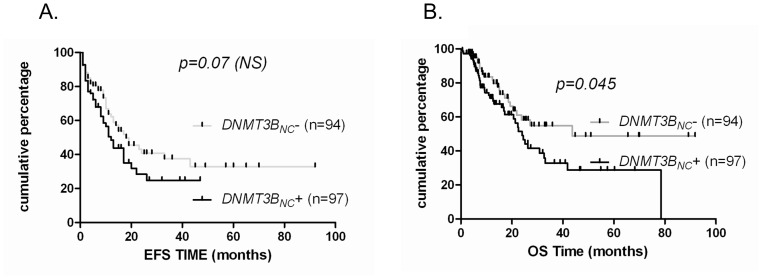
High DNMT*3B_NC_* expression associates with poor survival outcome. Kaplan-Meier analysis of two groups with expression levels above or below the cut-off as described in method section for *DNMT3B_NC_*. EFS (A) and OS (B) were assessed in 191 patients.

No significant differences were observed for the *DNMT3A* expression in the whole cohort population but its overexpression was related to a significant better EFS (p = 0.01) and OS (p = 0.012) in the normal karyotype AML subgroup. There was no association between *DNMT3A* exon 23 mutations and their prognostic impact among the whole population, patients with normal cytogenetic profiles or those with *FLT3ITD* mutations independently of age. Patients with an overexpression of *DNMT3B* and *3B_NC_* had worse EFS (p = 0.006) and OS (p = 0.045), respectively (Figure 6A and 7B) and a trend to a worse OS (p = 0.056) and to a worse EFS (p = 0.07), respectively (Figure 6B and 7A).

**Figure 8:**
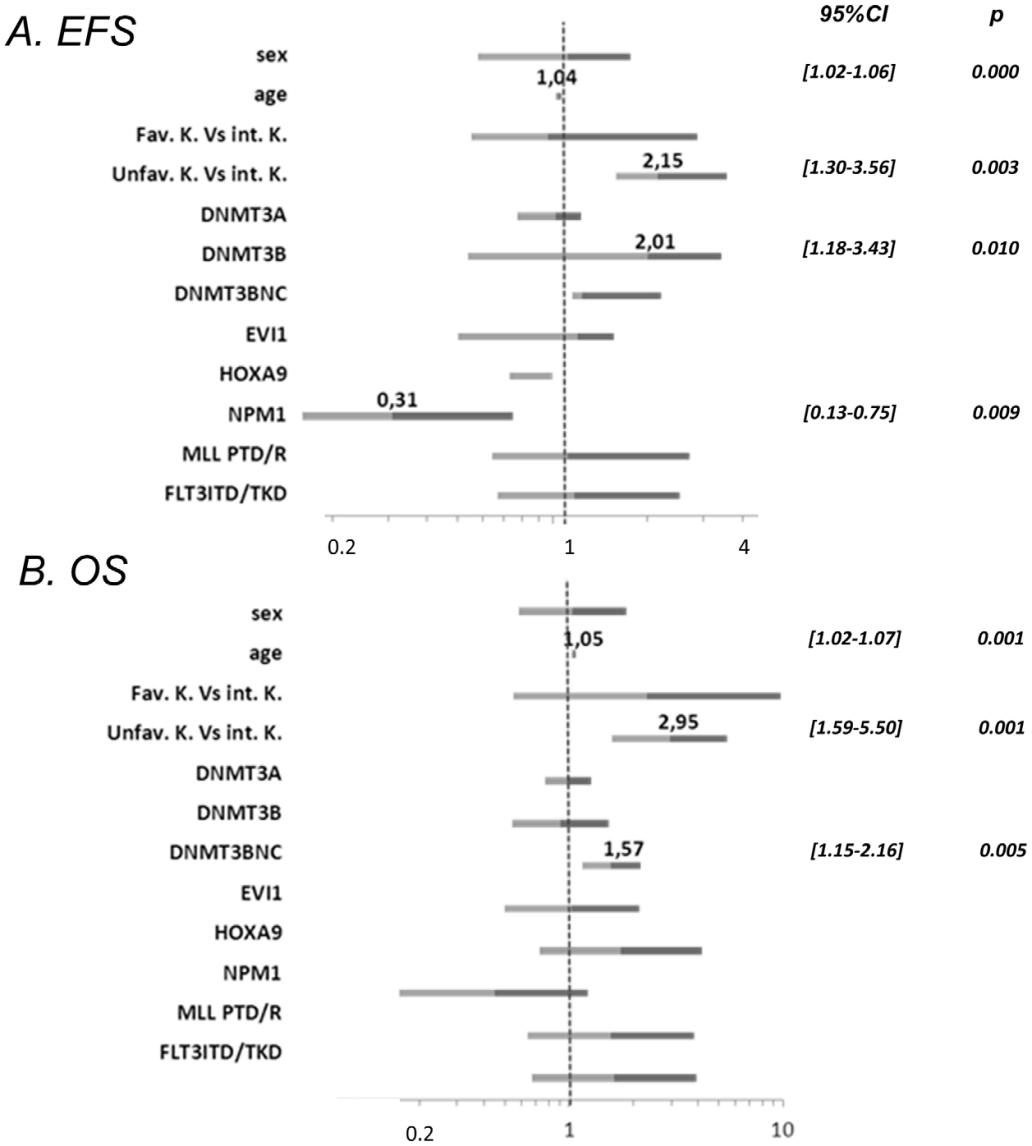
Multivariate analysis of High *DNMT3B/3B^NC^* expression as prognostic factors. Forest plot of multivariate analysis. Covariates tested in multivariate Cox models were sex, age, *DNMTs, HOXA9, EVI1* expressions, and cytogenetic subgroups: favourable karyotype (Fav. K.) and unfavourable karyotype (Unfav. K.) versus intermediate karyotype (int.K.) for (A) event free survival (EFS) and (B) overall survival (OS). P values were calculated using the Cox regression model, significant p values (p<0.05) are indicated Hazard Ratio are specified on the forest plot when p values are significant; CI, Confidence Interval (95%).

It should be noted that the prognostic impact of these markers was erased by the transplantation when considering the small cohort of patients allografted in first remission.Multivariate analysis showed a significant negative impact of age, unfavourable complex karyotype (more than 3 abnormalities, cytogenetic group 3) and *DNMT3B/B_NC_* levels on EFS and OS (Figure 8A and 8B). In addition we observed a positive impact of NPM1 mutations on EFS. Regarding the normal karyotype AML subgroup only a negative trend for the *DNMT3B* expression on EFS and OS (p = 0.07) was detected.

## Discussion

The deregulation of DNMT expression clearly contributes to tumorogenesis and its overexpression has been described as a poor prognostic marker in various malignancies [26], [27], [28]. Aberrant DNA hypermethylation feature has been directed by EVI1 in AML [1] which can suggest a strong relationship between these proteins. *EVI1* has been recognized as one of the most aggressive oncogene associated with AML [15], [16], [17]. Even if it has been recently shown in a large cohort of AML patients that its poor prognosis is independent from the *EVI1* spliceoform expressed [17], the poor impact of the *EVI1+/1D* has always been confirmed and should be relevant for stratifying patients in therapeutic protocols. Our results confirm that *EVI1* overexpression has an adverse prognostic impact, either in young or elderly AML patients. It is of note that *EVI1* is rarely overexpressed in mutated *NPM1* patients. An association between *EVI1* overexpression and *MLL* abnormalities has been previously noted by other [17]. EVI1 is frequently up-regulated in bone marrow cells transformed by the MLL oncoproteins and could be one of its targets which could explain their high association in leukemogenesis [42]. It has been previously described [41], that the total lack of *EVI1* expression might have a good prognosis, in our series this hypothesis was not confirmed.

A direct recruitment of DNMT3A and 3B by EVI1could reflect strong relationship between these proteins [1], [19]. In our series, *EVI1+* is not associated with high expression level of *DNMTs* at mRNA level which suggest functional interaction between these proteins rather than a common regulatory mechanism at their transcriptional levels. However, *EVI1+* is highly associated with absence of DNMT3A exon 23 mutations which could suggest a functional DNMT3A among *EVI1*+ patients. This hypothesis may be modulated by the identification of 3 mutated patients in the catalytic domain of DNMT3A. As previously described, we confirm here the high frequency of DNMT3A exon 23 mutations. These mutations were strongly associated to AML with a normal karyotype and with NPM1 mutations, and to a lower extend to those with FLT3 abnormalities and monocytic involvement. Moreover, we showed that DNMT3A exon 23 mutations were highly associated with *HOXA9* expression. The poor prognostic impact of DNMT3A mutations was not confirmed, probably due to the small number of patients [40] . Constitutional mutations in the catalytic domain of DNMT3B have been described and are responsible for hereditary syndrome characterized by ICF (immunodeficiency, instability of the centromeric region of chromosomes and facial abnormalities) in humans [43]. More recently, all tested ICF mutations are responsible of altered catalytic properties of DNMT3B [44]. No change was observed in the catalytic active variant of DNMT3B which could strongly suggest that mutation of DNMT3B may be not a common event In AML.

*DNMT3A* and *3B* transcripts seem to be inversely expressed in AML patients and related to *HOXA9* expression. *DNMT3B* and *3B_NC_* are highly related since they have the same promoter and are splicing variants. We have shown worse prognostic outcome associated with high *DNMT3B/3B_NC_* expression levels. The functions of DNMT3B_NC_ are not clearly defined. These truncated proteins, lacking the catalytic domain, could act as a rheostat in modulating DNMT3B and/or 3A. However they have been recently involved in various human tumours. Thus, forced expression of DNMT3B7, which could be the main isoform quantified in our assays, led to altered DNA methylation levels, particularly in hematologic malignancies. It has recently been shown in MYC-transgenic mice that overexpression of the non-catalytic Dnmt3b7 isoform or inactivation of the catalytically active Dnmt3b accelerated lymphomagenesis [14], [45]. Overexpression of catalytically inactive isoforms seems to have similar consequences as inactivation of active Dnmt3b isoforms and suggest possible oncogenic functions of catalytically inactive Dnmt3b isoforms, perhaps acting as dominant negative isoforms. In this context, we have tried to evaluate EFS and OS independently in cohorts of patients that had high active *DNMT3B* but low *DNMT3B_NC_* and *vice versa*. We did not find significant prognostic impact, probably related to the fact that *DNMT3B/3B_NC_* are highly correlated (p<0.0000) and therefore the number of patients in each cohort is too small (24 versus 18 patients, data not shown). In human AML, overexpression of *DNMT3B* and *3B_NC_* both seem to be correlated with poor prognosis and could participate together to the oncogenic methylation alterations in leukemic cells.

In spare cases, we have detected a significant prognostic impact of one molecular marker on EFS and not on OS (as *DNMT3B* which has shown only a trend to a worse OS) or *vice versa* (as *DNMT3BNC* or *HOXA9*). The clinical significance of these observations could be related to the efficiency of relapse treatments which improved OS, or conversely, related to very high mortality rates in first line setting that would principally affected OS and this, according to the expression of the considered marker.

We could not explain the relationship between *HOXA9* and *DNMT3* expressions or with *NPM1* mutations but homeotic proteins are largely involved in leukemogenesis, in MLL transduction pathway and more recently, one of this protein (HOXB3) has been shown to regulate DNMT3B expression in human cancer cell lines [46]. Nevertheless, we have shown that *DNMT3A* overexpression was associated with a favourable outcome in normal karyotype AML subgroup and *DNMT3B* (and *3B_NC_*) overexpression with a worse outcome. More recently DNMT3B expression level has been shown to be an adverse prognosis marker in diffuse large B-cell lymphomas [27]. In this setting, patients with DNMT overexpression were characterized by aggressive disease and poor prognosis, probably in relation to the hypermethylation of important genes in homeostasis although no target gene have been clearly identified in AML. In the future, these observed results could suggest that treatment specifically targeting methylation as cytosine analog drugs which interfere with methylation (5-azacytidine and decitabine (5-aza-20-deoxycytidine) should be clinically evaluated in those specific patients.

In conclusion this study represents the first report showing the prognostic impact of *DNMT3A*, *DNMT3B* and *3B_NC_* overexpression in AML. *DNMT3B (3B_NC_)* overexpression represents a new independent poor prognostic marker in AML. This should be useful for identifying patients who may benefit from demethylating agents.

## Supporting Information

Figure S1 **HRM analysis of DNMT3A exon 23 mutations. A.** HRM profiles of 8 patients (in duplicate) harbouring DNMT3A exon 23 mutations (red) compared to 20 negative patients (blue). **B.** The sensitivity of the test (∼ 2.5%) was obtained from the dilution of R882H mutated samples in non mutated cDNA (undiluted: heterozygous rate at 50%); Analysis was performed on the LC480 Roche device. Positive detected dilutions in red.
(TIF)

## References

[1] Lugthart S, Figueroa ME, Bindels E, Skrabanek L, Valk PJ, et al (2011) Aberrant DNA hypermethylation signature in acute myeloid leukemia directed by EVI1. Blood 117: 234–241. 20855866 10.1182/blood-2010-04-281337 PMC3037746

[2] Figueroa ME, Lugthart S, Li Y, Erpelinck-Verschueren C, Deng X, et al (2010) DNA methylation signatures identify biologically distinct subtypes in acute myeloid leukemia. Cancer Cell 17: 13–27. 20060365 10.1016/j.ccr.2009.11.020 PMC3008568

[3] Razin A, Riggs AD (1980) DNA methylation and gene function. Science 210: 604–610. 6254144 10.1126/science.6254144

[4] Robertson KD (2001) DNA methylation, methyltransferases, and cancer. Oncogene 20: 3139–3155. 11420731 10.1038/sj.onc.1204341

[5] Arand J, Spieler D, Karius T, Branco MR, Meilinger D, et al (2012) In Vivo Control of CpG and Non-CpG DNA Methylation by DNA Methyltransferases. PLoS Genet 8: e1002750. 22761581 10.1371/journal.pgen.1002750 PMC3386304

[6] Okano M, Bell DW, Haber DA, Li E (1999) DNA methyltransferases Dnmt3a and Dnmt3b are essential for de novo methylation and mammalian development. Cell 99: 247–257. 10555141 10.1016/s0092-8674(00)81656-6

[7] Chen T, Tsujimoto N, Li E (2004) The PWWP domain of Dnmt3a and Dnmt3b is required for directing DNA methylation to the major satellite repeats at pericentric heterochromatin. Mol Cell Biol 24: 9048–9058. 15456878 10.1128/MCB.24.20.9048-9058.2004 PMC517890

[8] Kaneda M, Okano M, Hata K, Sado T, Tsujimoto N, et al (2004) Essential role for de novo DNA methyltransferase Dnmt3a in paternal and maternal imprinting. Nature 429: 900–903. 15215868 10.1038/nature02633

[9] Ostler KR, Davis EM, Payne SL, Gosalia BB, Exposito-Cespedes J, et al (2007) Cancer cells express aberrant DNMT3B transcripts encoding truncated proteins. Oncogene 26: 5553–5563. 17353906 10.1038/sj.onc.1210351 PMC2435620

[10] Van Emburgh BO, Robertson KD (2011) Modulation of Dnmt3b function in vitro by interactions with Dnmt3L, Dnmt3a and Dnmt3b splice variants. Nucleic Acids Res 39: 4984–5002. 21378119 10.1093/nar/gkr116 PMC3130282

[11] Jones PA, Baylin SB (2007) The epigenomics of cancer. Cell 128: 683–692. 17320506 10.1016/j.cell.2007.01.029 PMC3894624

[12] Figueroa ME, Abdel-Wahab O, Lu C, Ward PS, Patel J, et al (2010) Leukemic IDH1 and IDH2 mutations result in a hypermethylation phenotype, disrupt TET2 function, and impair hematopoietic differentiation. Cancer Cell 18: 553–567. 21130701 10.1016/j.ccr.2010.11.015 PMC4105845

[13] Ostler KR, Yang Q, Looney TJ, Zhang L, Vasanthakumar A, et al (2012) Truncated DNMT3B Isoform DNMT3B7 Suppresses Growth, Induces Differentiation, and Alters DNA Methylation in Human Neuroblastoma. Cancer Res 72: 4714–4723. 22815530 10.1158/0008-5472.CAN-12-0886 PMC3445765

[14] Shah MY, Vasanthakumar A, Barnes NY, Figueroa ME, Kamp A, et al (2010) DNMT3B7, a truncated DNMT3B isoform expressed in human tumors, disrupts embryonic development and accelerates lymphomagenesis. Cancer Res 70: 5840–5850. 20587527 10.1158/0008-5472.CAN-10-0847 PMC2905468

[15] Nucifora G, Laricchia-Robbio L, Senyuk V (2006) EVI1 and hematopoietic disorders: history and perspectives. Gene 368: 1–11. 16314052 10.1016/j.gene.2005.09.020

[16] Lugthart S, van Drunen E, van Norden Y, van Hoven A, Erpelinck CA, et al (2008) High EVI1 levels predict adverse outcome in acute myeloid leukemia: prevalence of EVI1 overexpression and chromosome 3q26 abnormalities underestimated. Blood 111: 4329–4337. 18272813 10.1182/blood-2007-10-119230

[17] Groschel S, Lugthart S, Schlenk RF, Valk PJ, Eiwen K, et al (2010) High EVI1 expression predicts outcome in younger adult patients with acute myeloid leukemia and is associated with distinct cytogenetic abnormalities. J Clin Oncol 28: 2101–2107. 20308656 10.1200/JCO.2009.26.0646

[18] Wieser R (2007) The oncogene and developmental regulator EVI1: expression, biochemical properties, and biological functions. Gene 396: 346–357. 17507183 10.1016/j.gene.2007.04.012

[19] Senyuk V, Premanand K, Xu P, Qian Z, Nucifora G (2011) The oncoprotein EVI1 and the DNA methyltransferase Dnmt3 co-operate in binding and de novo methylation of target DNA. PLoS One 6: e20793. 21695170 10.1371/journal.pone.0020793 PMC3112226

[20] Walter MJ, Ding L, Shen D, Shao J, Grillot M, et al (2011) Recurrent DNMT3A mutations in patients with myelodysplastic syndromes. Leukemia 25: 1153–1158. 21415852 10.1038/leu.2011.44 PMC3202965

[21] Thol F, Winschel C, Ludeking A, Yun H, Friesen I, et al (2011) Rare occurrence of DNMT3A mutations in myelodysplastic syndromes. Haematologica 96: 1870–1873. 21880636 10.3324/haematol.2011.045559 PMC3232272

[22] Ley TJ, Ding L, Walter MJ, McLellan MD, Lamprecht T, et al (2010) DNMT3A mutations in acute myeloid leukemia. N Engl J Med 363: 2424–2433. 21067377 10.1056/NEJMoa1005143 PMC3201818

[23] Renneville A, Boissel N, Nibourel O, Berthon C, Helevaut N, et al.. (2012) Prognostic significance of DNA methyltransferase 3A mutations in cytogenetically normal acute myeloid leukemia: a study by the Acute Leukemia French Association. Leukemia: Epub ahead of print. 10.1038/leu.2011.382 22289988

[24] Thol F, Damm F, Ludeking A, Winschel C, Wagner K, et al (2011) Incidence and prognostic influence of DNMT3A mutations in acute myeloid leukemia. J Clin Oncol 29: 2889–2896. 21670448 10.1200/JCO.2011.35.4894

[25] Ribeiro AF, Pratcorona M, Erpelinck-Verschueren C, Rockova V, Sanders M, et al (2012) Mutant DNMT3A: a marker of poor prognosis in acute myeloid leukemia. Blood 119: 5824–5831. 22490330 10.1182/blood-2011-07-367961

[26] Wang J, Walsh G, Liu DD, Lee JJ, Mao L (2006) Expression of Delta DNMT3B variants and its association with promoter methylation of p16 and RASSF1A in primary non-small cell lung cancer. Cancer Res 66: 8361–8366. 16951144 10.1158/0008-5472.CAN-06-2031

[27] Amara K, Ziadi S, Hachana M, Soltani N, Korbi S, et al (2010) DNA methyltransferase DNMT3b protein overexpression as a prognostic factor in patients with diffuse large B-cell lymphomas. Cancer Sci 101: 1722–1730. 20398054 10.1111/j.1349-7006.2010.01569.x PMC11159814

[28] Zhang JJ, Zhu Y, Zhu Y, Wu JL, Liang WB, et al (2012) Association of increased DNA methyltransferase expression with carcinogenesis and poor prognosis in pancreatic ductal adenocarcinoma. Clin Transl Oncol 14: 116–124. 22301400 10.1007/s12094-012-0770-x

[29] Bennett JM, Catovsky D, Daniel MT, Flandrin G, Galton DA, et al (1976) Proposals for the classification of the acute leukaemias. French-American-British (FAB) co-operative group. Br J Haematol 33: 451–458. 188440 10.1111/j.1365-2141.1976.tb03563.x

[30] Arber DA Brunning RD, Le Beau MM, Falini B (2008) Acute myeloid leukemia with recurrent genetic abnormalities In: Swerdlow SH, Harris NE, Pileri SA, Stein H, Thiele J, et al., editors. WHO classification of tumours of haematopoietic and lymphoid tissues Lyon, France: International Agency for reseach on cancer Press (IARC). 110–123.

[31] Grimwade D, Walker H, Oliver F, Wheatley K, Harrison C, et al (1998) The importance of diagnostic cytogenetics on outcome in AML: analysis of 1,612 patients entered into the MRC AML 10 trial. The Medical Research Council Adult and Children's Leukaemia Working Parties. Blood 92: 2322–2333. 9746770

[32] Boissel N, Cayuela JM, Preudhomme C, Thomas X, Grardel N, et al (2002) Prognostic significance of FLT3 internal tandem repeat in patients with de novo acute myeloid leukemia treated with reinforced courses of chemotherapy. Leukemia 16: 1699–1704. 12200684 10.1038/sj.leu.2402622

[33] Boissel N, Renneville A, Biggio V, Philippe N, Thomas X, et al (2005) Prevalence, clinical profile, and prognosis of NPM mutations in AML with normal karyotype. Blood 106: 3618–3620. 16046528 10.1182/blood-2005-05-2174

[34] Poirel H, Rack K, Delabesse E, Radford-Weiss I, Troussard X, et al (1996) Incidence and characterization of MLL gene (11q23) rearrangements in acute myeloid leukemia M1 and M5. Blood 87: 2496–2505. 8630416

[35] Barjesteh van Waalwijk van Doorn-Khosrovani S, Erpelinck C, van Putten WL, Valk PJ, van der Poel-van de Luytgaarde S, et al (2003) High EVI1 expression predicts poor survival in acute myeloid leukemia: a study of 319 de novo AML patients. Blood 101: 837–845. 12393383 10.1182/blood-2002-05-1459

[36] Gabert J, Beillard E, van der Velden VH, Bi W, Grimwade D, et al (2003) Standardization and quality control studies of 'real-time' quantitative reverse transcriptase polymerase chain reaction of fusion gene transcripts for residual disease detection in leukemia - a Europe Against Cancer program. Leukemia 17: 2318–2357. 14562125 10.1038/sj.leu.2403135

[37] Weisser M, Haferlach T, Schoch C, Hiddemann W, Schnittger S (2004) The use of housekeeping genes for real-time PCR-based quantification of fusion gene transcripts in acute myeloid leukemia. Leukemia 18: 1551–1553. 15284861 10.1038/sj.leu.2403438

[38] Cheson BD, Bennett JM, Kopecky KJ, Buchner T, Willman CL, et al (2003) Revised recommendations of the International Working Group for Diagnosis, Standardization of Response Criteria, Treatment Outcomes, and Reporting Standards for Therapeutic Trials in Acute Myeloid Leukemia. J Clin Oncol 21: 4642–4649. 14673054 10.1200/JCO.2003.04.036

[39] Thomas X, Elhamri M, Raffoux E, Renneville A, Pautas C, et al (2011) Comparison of high-dose cytarabine and timed-sequential chemotherapy as consolidation for younger adults with AML in first remission: the ALFA-9802 study. Blood 118: 1754–1762. 21690555 10.1182/blood-2011-04-349258

[40] Larochelle O, Bertoli S, Vergez F, Sarry JE, Mansat-De Mas V, et al (2011) Do AML patients with DNMT3A exon 23 mutations benefit from idarubicin as compared to daunorubicin? A single center experience. Oncotarget 2: 850–861. 22081665 10.18632/oncotarget.347 PMC3260002

[41] Vazquez I, Maicas M, Cervera J, Agirre X, Marin-Bejar O, et al (2011) Down-regulation of EVI1 is associated with epigenetic alterations and good prognosis in patients with acute myeloid leukemia. Haematologica 96: 1448–1456. 21750091 10.3324/haematol.2011.040535 PMC3186305

[42] Arai S, Yoshimi A, Shimabe M, Ichikawa M, Nakagawa M, et al (2011) Evi-1 is a transcriptional target of mixed-lineage leukemia oncoproteins in hematopoietic stem cells. Blood 117: 6304–6314. 21190993 10.1182/blood-2009-07-234310

[43] Ehrlich M (2003) The ICF syndrome, a DNA methyltransferase 3B deficiency and immunodeficiency disease. Clin Immunol 109: 17–28. 14585272 10.1016/s1521-6616(03)00201-8

[44] Moarefi AH, Chedin F (2011) ICF syndrome mutations cause a broad spectrum of biochemical defects in DNMT3B-mediated de novo DNA methylation. J Mol Biol 409: 758–772. 21549127 10.1016/j.jmb.2011.04.050

[45] Hlady RA, Novakova S, Opavska J, Klinkebiel D, Peters SL, et al (2012) Loss of Dnmt3b function upregulates the tumor modifier Ment and accelerates mouse lymphomagenesis. J Clin Invest 122: 163–177. 22133874 10.1172/JCI57292 PMC3248285

[46] Palakurthy RK, Wajapeyee N, Santra MK, Gazin C, Lin L, et al (2009) Epigenetic silencing of the RASSF1A tumor suppressor gene through HOXB3-mediated induction of DNMT3B expression. Mol Cell 36: 219–230. 19854132 10.1016/j.molcel.2009.10.009 PMC2776651

