# Supplementary figures and images for: High DNA Methyltransferase DNMT3B Levels: A Poor Prognostic Marker in Acute Myeloid Leukemia
Source: PLoS One. 2012 Dec 10;7(12):e51527. doi: 10.1371/journal.pone.0051527 (PMC3519733; doi:10.1371/journal.pone.0051527)

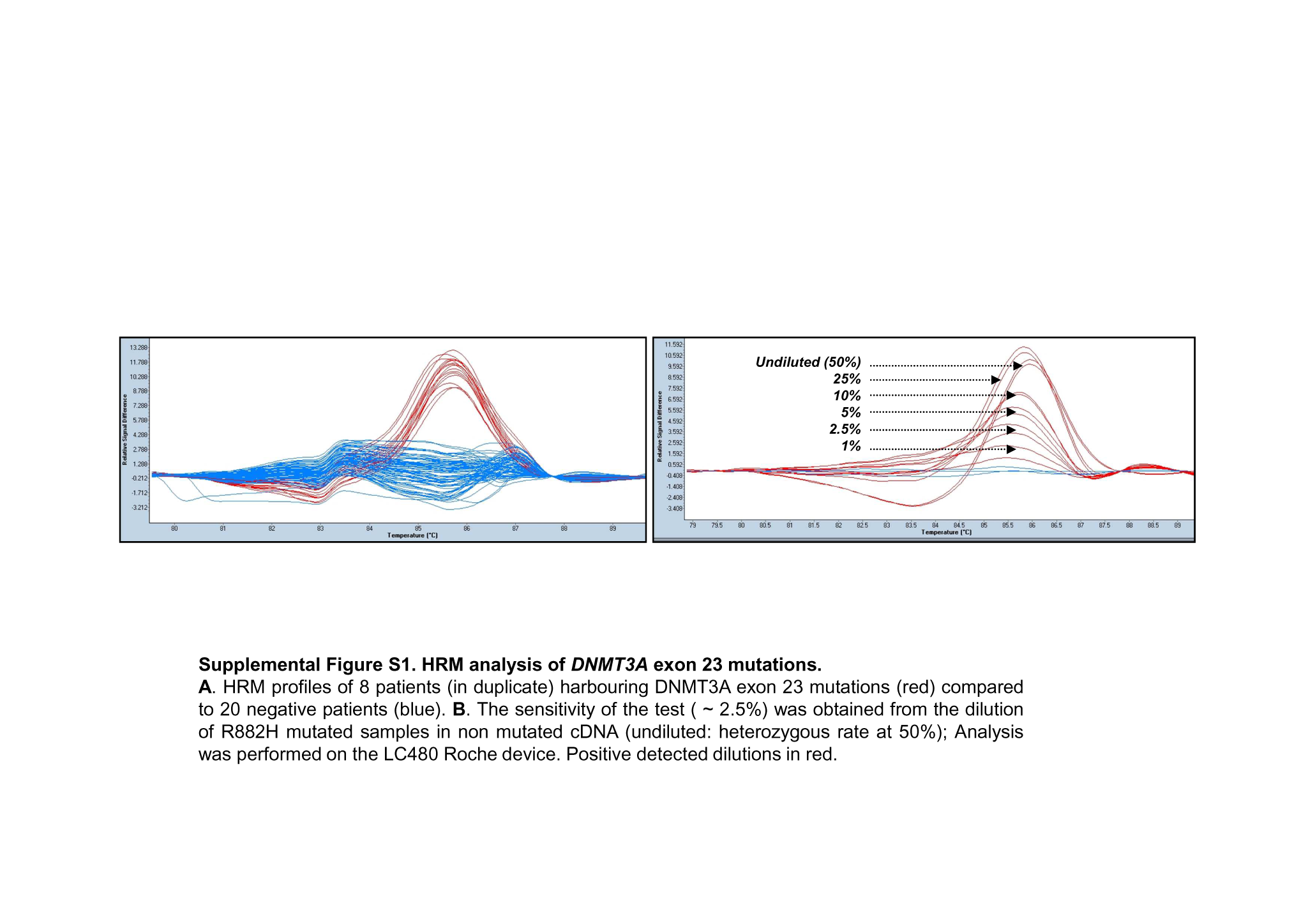

Supplement: Figure S1 — HRM analysis of DNMT3A exon 23 mutations. A. HRM profiles of 8 patients (in duplicate) harbouring DNMT3A exon 23 mutations (red) compared to 20 negative patients (blue). B. The sensitivity of the test (∼ 2.5%) was obtained from the dilution of R882H mutated samples in non mutated cDNA (undiluted: heterozygous rate at 50%); Analysis was performed on the LC480 Roche device. Positive detected dilutions in red. (TIF) [file pone.0051527.s001.tif]
